# Associations of Five Insulin Resistance‐Related Indices With Gout Risk in the 2007–2018 NHANES Cross‐Sectional Study

**DOI:** 10.1155/ije/7431198

**Published:** 2026-08-29

**Authors:** Kewei Yang, Kongming Yang, Yuhan Liu, Qin Wang, Nan Zhang, Yuanping Yin

**Affiliations:** ^1^ Liaoning University of Traditional Chinese Medicine, Shenyang, China, lnutcm.edu.cn; ^2^ Liaoning University of Traditional Chinese Medicine Affiliated Second Hospital, Shenyang, China

**Keywords:** central obesity, cross-sectional study, gout, insulin resistance, metabolic syndrome

## Abstract

**Objective:**

This study investigated the associations between five insulin resistance (IR)‐related surrogate indices and gout and compared their associations and discriminative abilities for gout. The indices included the triglyceride‐glucose (TyG) index, TyG combined with body mass index (TyG‐BMI), lipid accumulation product (LAP), visceral adiposity index (VAI), and metabolic score for insulin resistance (METS‐IR).

**Methods:**

Data from the 2007–2018 National Health and Nutrition Examination Survey (NHANES) were analyzed. Comparisons between the gout and nongout groups were performed using *t*‐tests and chi‐square tests. Multivariable logistic regression and subgroup analyses were used to assess the associations.

**Results:**

Among 14,582 participants (4.80% with gout), adjusted analyses identified the highest METS‐IR quartile as the strongest predictor of gout (adjusted OR = 2.823, 95%CI 1.643–4.851), followed by TyG‐BMI (OR = 2.290, 95%CI 1.555–3.373). Restricted cubic splines revealed nonlinear associations of TyG and LAP with gout risk, contrasting with linear trends for TyG‐BMI, VAI, and METS‐IR. Subgroup analyses suggested that elevated TyG and VAI were positively associated with gout in nondiabetic individuals (interaction *p* < 0.05).

**Conclusion:**

All five IR‐related indices showed positive associations with gout, particularly in those with central obesity. These indices showed modest discriminative ability for gout, and further validation is needed.

## 1. Introduction

Gout is an inflammatory joint disease caused by urate crystal deposition and has a global incidence of 1%–6.8% [1]. It is also associated with obesity and metabolic syndrome [2]. Patients with early‐stage disease are often asymptomatic but are at risk of complications such as arthritis, nephropathy, hypertension, and diabetes mellitus [3, 4]. Given its prevalence and health effects [5], gout is a major public health issue that requires early prevention and treatment.

As an important representative of metabolic diseases, gout shares a core pathological mechanism with diabetes through insulin resistance (IR) [6–8]. Studies have shown that IR leads to decreased glucose uptake by peripheral tissues through dysregulation of signaling pathways, such as IRS‐PI3K‐AKT/mTOR, and inhibits renal excretion of uric acid, resulting in a vicious cycle of hyperglycemia and hyperuricemia [9, 10]. Clinical studies have further revealed that hyperuricemia exacerbates IR by inhibiting insulin signaling, while hyperinsulinemia in the IR state reduces uric acid excretion, forming a metabolic “iron triangle.” Recent intervention trials have demonstrated that uric acid‐lowering drugs such as febuxostat can improve IR indices [11], suggesting bidirectional regulation. These pathway mechanisms provide molecular targets for the management of diabetes and gout comorbidities.

To assess IR, various noninsulin‐based indices have been developed, including the triglyceride glucose index (TyG) [12, 13], TyG combined with body mass index (TyG‐BMI) [14], visceral adiposity index (VAI) [15], lipid accumulation product (LAP) [16], and metabolic score for insulin resistance (METS‐IR) [17]. These indices are gaining attention in clinical practice due to their simplicity and affordability.

Recent observational studies have identified hyperuricemia as an independent associated factor for IR and prediabetes [18]; however, these findings may have reverse causation or residual confounding effects. Some human physiological experiments have shown [19] that secondary hyperinsulinemia can increase serum uric acid (SUA) concentrations; however, its population‐level effects are unknown. The relationship between the TyG index and gouty arthritis has been reported in observational studies [20, 21], but few studies have examined the dose–response relationship between the TyG index and gout risk or compared different IR substitutes in relation to gout. Based on the established link between IR and gout, this study evaluated five IR‐related indices (TyG, TyG‐BMI, LAP, VAI, and METS‐IR) and was conducted in a US population to characterize the correlation between these proxies and the prevalence of gout, thereby facilitating the identification of individuals at higher risk for gout.

## 2. Materials and Methods

### 2.1. Study Population

**FIGURE 1:**
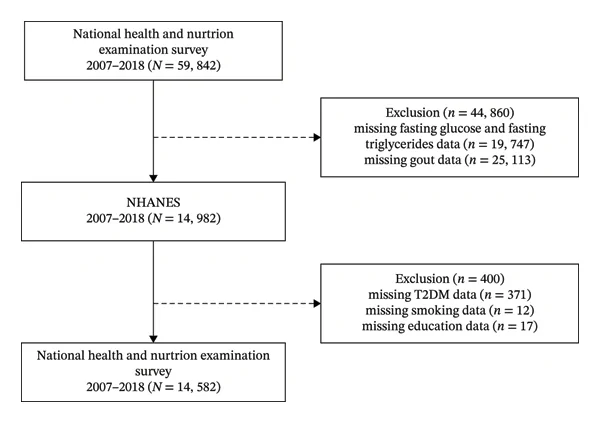
Flowchart of participant selection from NHANES (2007–2018).

This study utilized data from the National Health and Nutrition Examination Survey (NHANES), a cross‐sectional, nationally representative survey designed to assess the health and nutritional status of the noninstitutionalized civilian population in the United States. A total of 59,842 individuals from six consecutive 2‐year NHANES cycles (2007–2018) were considered for inclusion. The exclusion criteria were as follows: (1) missing data on gout (*n* = 25,113), (2) incomplete records on fasting glucose and fasting TGs (*n* = 19,747), (3) absence of T2DM records (*n* = 371), (4) missing data on cigarette smoking (*n* = 12), and (5) missing information on education (*n* = 17). After applying these criteria, data from 14,582 participants were analyzed (Figure 1). The study protocols were approved by the Institutional Review Board of the National Center for Health Statistics (NCHS), and written informed consent was obtained from all participants.

### 2.2. Exposure

Blood was collected in the morning after fasting to measure the high‐density lipoprotein cholesterol (HDL‐C), low‐density lipoprotein cholesterol (LDL‐C), TGs, fasting glucose, and insulin levels. The exposures of interest were five IR proxies, TyG, TyG‐BMI, LAP, VAI, and METS‐IR, which were calculated using the following equations:

 TyG = ln [TG (mg/dL) × FPG (mg/dL)/2]; [22].
 TyG‐BMI = TyG × BMI; [14].
 LAP = (WC‐65) × TG (men), LAP = (WC‐58) × TG (women); [23].
 VAI = (WC/(39.68 + (1.88 × BMI))) × (TG/1.03) × (1.31/HDL‐C) (men),
 VAI = (WC/(36.58 + (1.89 × BMI))) × (TG/0.81) × (1.31/HDL‐C) (women); [15].
 METS‐IR = ln [(2 × FPG (mg/dL)) + TG (mg/dL)] × BMI)/(Ln [HDL‐C (mg/dL)]); [24].

Participants were categorized into four groups (Q1, Q2, Q3, and Q4) according to the quartiles of the index, with Q1 as the reference group.

### 2.3. Assessment of Gout

Similar to previous NHANES reports [25–27], the determination of gout status among participants was based on self‐reported data collected through a structured questionnaire administered by trained NHANES interviewers. During the medical conditions interview, participants were asked the following question: “Has a doctor or other health professional ever told you that you had gout?” Participants who responded affirmatively to this question were classified as having gout, whereas those who responded negatively were categorized as not having gout. This binary classification served as the primary outcome variable for subsequent analyses.

### 2.4. Covariates

Demographics included age ≥ 20 years, sex (female/male), race, ethnicity (Mexican American, non‐Hispanic Black, non‐Hispanic White, other Hispanic, other race—including multiracial), and education level (less than 9th grade, high school graduate/GED or equivalent, some college or AA degree, college graduate or above, 9–11th grade [including 12th grade with no diploma]), and the family income‐to‐poverty ratio (PIR). According to the literature [28–30], several other covariates were included in this study: alcohol consumption, folic acid, smoking, and diabetes mellitus. Diabetes mellitus was ascertained using a composite criterion. Individuals were categorized as having diabetes if they reported a prior clinical diagnosis by a healthcare professional, were currently taking glucose‐lowering agents, or exhibited abnormal glycemic markers. Specifically, the laboratory thresholds included glycated hemoglobin (HbA1c) ≥ 6.5%, fasting plasma glucose ≥ 7.0 mmol/L, or random plasma glucose ≥ 11.1 mmol/L. SUA, fasting insulin, TG, LDL‐C, and HDL‐C levels were measured clinically in the NHANES laboratory.

### 2.5. Statistical Analysis

To ensure national representativeness, we combined six NHANES cycles spanning 2007–2008 through 2017–2018. Given that the two‐year fasting subsample weights are cycle‐specific, we divided these weights by six to generate 12‐year weights, which were then applied across all analyses. The complex survey design was accounted for by incorporating the stratification variable (SDMVSTRA) and primary sampling unit (SDMVPSU) using the svydesign function in R. All analyses were restricted to participants with available fasting subsample weights to ensure appropriate application of the fasting‐specific weighting scheme. This weighting approach ensured that our estimates remained representative of the noninstitutionalized U.S. civilian population over the combined study period. Participant characteristics are presented as 95% confidence interval means for continuous variables and percentage frequencies for categorical variables using *t*‐tests and *x* ^2^ tests. Multivariate logistic regression analysis was used to assess the association between the five IR‐related proxies and gout. Following the STROBE (Strengthening the Reporting of Observational Studies in Epidemiology, the details are in the supporting information 1, STROBE‐checklist‐v4‐cross‐sectional) guidelines, four models were constructed: Model 1, without covariate adjustment; Model 2, adjusted for age, sex, race, education level, and household income to poverty ratio; Model 3, Model 2 plus smoking, alcohol consumption, uric acid, and folic acid; and Model 4, Model 3 plus diabetes, fasting insulin, TGs, LDL‐C, and HDL‐C. In this analysis, diabetes was assigned dual roles based on its established interconnections with hyperuricemia and gout. First, it was included as a covariate in the primary multivariate models to control for potential confounding, given that diabetes is associated with both SUA levels and gout risk via renal handling and metabolic factors. Second, we performed stratified analyses by diabetes status to evaluate its potential effect modification, as the underlying metabolic and inflammatory milieu may substantially differ between diabetic and nondiabetic individuals. To address concerns about potential attenuation of associations due to adjustment for variables along the IR–gout pathway (TGs, HDL‐C, fasting insulin, and uric acid), we conducted a sensitivity analysis excluding these variables from the covariate adjustment while additionally adjusting for kidney function parameters (eGFR and serum creatinine). Box‐and‐line plots were used to explore distributional differences between gout status and index levels, restricted cubic spline curves were used to assess nonlinear associations, and threshold effect analyses were used to identify possible critical values. The log‐likelihood ratio test was used to evaluate interactions between the indices and subgroup variables. Finally, this study compared the discriminatory ability of gout with different IR replacement indices using the receiver operating characteristic (ROC) curves and area under the curve (AUC), with differences compared using the *Z*‐test.

This study was analyzed using the R software package (R Foundation for Statistical Computing, Vienna, Austria, Version 4.4.2) and Empowerment software (https://www.empowerstats.net). Statistical significance was set at *p* < 0.05.

## 3. Results

### 3.1. Characteristics of Study Participants

A total of 14,582 participants were analyzed, of whom 700 (4.8%) had gout. Table 1 shows the baseline characteristics of patients with and without gout. Continuous variables were expressed as mean (standard error), and categorical variables as numbers and percentages. No significant differences were found in household income, poverty ratio, educational level, or alcohol consumption (all *p* > 0.05). The gout group had a mean age of 63.71 years; 69.1%, 48.7%, and 33.9% were men, non‐Hispanic whites, and diabetics, respectively (all *p* < 0.05). Biochemical parameters showed significant differences in folate, uric acid, insulin, TG, HDL‐C, and LDL‐C levels between groups (all *p* < 0.05). IR‐related indices, including the TyG index, TyG‐BMI, VAI, LAP, and METS‐IR, were significantly higher in patients with gout (*p* < 0.05).

**TABLE 1:** The characteristics of participants with gout.

Characteristics	Total (*n* = 14,582)	Nongout (*n* = 13,882)	Gout (*n* = 700)	*p* value
Gender (%)				< 0.001
Female	7541 (51.7)	7325 (52.8)	216 (30.9)	
Male	7041 (48.3)	6557 (47.2)	484 (69.1)	
Age (years)	49.88 (17.66)	49.18 (17.57)	63.71 (13.20)	< 0.001
Race (%)				< 0.001
Mexican American	2247 (15.4)	2190 (15.8)	57 (8.1)	
Non‐Hispanic Black	2908 (19.9)	2741 (19.7)	167 (23.9)	
Non‐Hispanic White	6046 (41.5)	5705 (41.1)	341 (48.7)	
Other Hispanic	1594 (10.9)	1544 (11.1)	50 (7.1)	
Other race—including multiracial	1787 (12.3)	1702 (12.3)	85 (12.1)	
Education (%)				0.243
Less than 9th grade	1583 (10.9)	1501 (10.8)	82 (11.7)	
9–11th grade	2088 (14.3)	1989 (14.3)	99 (14.1)	
High school graduate	3282 (22.5)	3109 (22.4)	173 (24.7)	
Some college or AA degree	4218 (28.9)	4013 (28.9)	205 (29.3)	
College graduate or above	3411 (23.4)	3270 (23.6)	141 (20.1)	
PIR	2.46 (1.62)	2.46 (1.62)	2.51 (1.60)	0.389
Smoke (%)				< 0.001
No	8129 (55.7)	7831 (56.4)	298 (42.6)	
Yes	6453 (44.3)	6051 (43.6)	402 (57.4)	
Alcohol drinks (day)	4.02 (35.67)	3.94 (34.55)	5.59 (53.28)	0.234
Folic acid (mcg)	173.39 (173.20)	174.10 (174.40)	159.29 (146.87)	0.027
Uric acid (µmol/L)	326.63 (85.91)	323.10 (82.99)	396.60 (109.71)	< 0.001
Diabetes (%)				< 0.001
No	12,563 (86.2)	12,100 (87.2)	463 (66.1)	
Yes	2019 (13.8)	1782 (12.8)	237 (33.9)	
Insulin (pmol/L)	83.98 (107.79)	82.46 (105.66)	114.11 (140.34)	< 0.001
HDL‐C (mg/dL)	53.94 (16.10)	54.15 (16.12)	49.77 (15.16)	< 0.001
TG (mg/dL)	124.95 (109.64)	123.61 (108.69)	151.67 (124.18)	< 0.001
LDL‐C (mg/dL)	113.25 (35.68)	113.59 (35.51)	106.52 (38.42)	< 0.001
TyG	8.61 (0.68)	8.60 (0.68)	8.92 (0.71)	< 0.001
TyG‐BMI	251.61 (67.42)	250.01 (66.63)	283.31 (74.79)	< 0.001
VAI	2.85 (2.71)	2.84 (2.68)	3.13 (3.17)	0.005
LAP	56.84 (61.97)	55.54 (60.27)	82.69 (85.24)	< 0.001
METS‐IR	43.32 (12.59)	43.02 (12.42)	49.33 (14.31)	< 0.001

*Note:* METS‐IR, metabolic score for IR.

Abbreviations: PIR, family income‐to‐poverty ratio; HDL‐C, high‐density lipoprotein cholesterol; TG, triglyceride; LDL‐C, low‐density lipoprotein cholesterol; TyG, triglyceride‐glucose index; TyG‐BMI, TyG index with body mass index; VAI, visceral adiposity index; LAP, lipid accumulation product.

### 3.2. Association Between IR Substitutes and Gout

In this study, we employed a weighted logistic regression model to evaluate the relationship between gout and indices related to IR across the four different models (Supporting Table 1). As shown in Table 2, the five IR‐related indices indicated a positive association with gout both before and after adjustments in the various models. An upward trend in the risk of gout was observed as the quartiles of TyG, TyG‐BMI, LAP, VAI, and METS‐IR increased, with METS‐IR showing the strongest link to gout. When examining the odds ratios (ORs), Model 4 revealed that the highest adjusted quartile of METS‐IR had the most pronounced impact on gout, with an OR of 2.823 and a 95% confidence interval (CI) of 1.643–4.851. This was followed by TyG‐BMI (), LAP (OR 1.902, 95% CI 1.219–2.968), VAI (OR 1.747, 95% CI 1.040–2.935), and TyG (OR 1.445, 95% CI 0.856–2.439). Although Model 4 indicated the best model fit based on Variance Inflation Factor (VIF) and AIC (Akaike Information Criterion) diagnostics (Supporting Tables 2–3), it includes variables that are conceptually related to the IR indices. Therefore, estimates from Model 4 should be interpreted with caution. In the sensitivity analysis (Supporting Table 4), excluding pathway variables (TGs, HDL‐C, fasting insulin, and uric acid) and additionally adjusting for diabetes, LDL‐C, eGFR, and serum creatinine, all five IR indices remained positively and dose–responsively associated with gout. The Q4 ORs were as follows: TyG, 2.120 (1.364–3.295); TyG‐BMI, 3.912 (2.788–5.488); VAI, 2.229 (1.550–3.206); LAP, 3.330 (2.254–4.921); and METS‐IR, 3.577 (2.328–5.496). These estimates were largely consistent with the fully adjusted model, indicating that adjustment for metabolic intermediates did not substantially alter the observed associations.

**TABLE 2:** Association between five IR‐related indices and gout patients.

Variables	OR (95% CI)			
	Model 1	Model 2	Model 3	Model 4
TyG				
Q1	Ref.			
Q2	1.310 (0.848–2.021)	1.084 (0.614–1.913)	0.988 (0.533–1.763)	1.012 (0.571–1.794)
Q3	2.063 (1.360–3.128)	1.623 (0.939–2.803)	1.334 (0.744–2.391)	1.306 (0.746–2.288)
Q4	3.307 (2.181–5.013)	2.477 (1.455–4.218)	1.794 (1.027–3.132)	1.445 (0.856–2.439)
TyG‐BMI				
Q1	Ref.			
Q2	1.920 (1.134–2.764)	1.268 (0.834–1.927)	1.133 (0.745–1.723)	1.137 (0.767–1.687)
Q3	2.676 (1.903–3.763)	2.113 (1.282–3.483)	1.676 (0.997–2.818)	1.599 (0.965–2.650)
Q4	4.512 (3.240–6.284)	3.833 (2.492–5.895)	2.738 (1.725–4.345)	2.290 (1.555–3.373)
VAI				
Q1	Ref.			
Q2	0.974 (0.716–1.3266)	1.364 (0.849–2.191)	1.205 (0.729–1.991)	1.071 (0.670–1.714)
Q3	0.968 (0.688–1.360)	1.955 (1.288–2.969)	1.597 (1.049–2.433)	1.363 (0.823–2.258)
Q4	1.406 (1.048–1.886)	2.832 (1.784–4.498)	2.108 (1.289–3.449)	1.747 (1.040–2.935)
LAP				
Q1	Ref.			
Q2	1.941 (1.325–2.842)	1.199 (0.750–1.918)	1.085 (0.675–1.744)	1.109 (0.697–1.765)
Q3	3.194 (2.202–4.633)	2.249 (2.376–3.678)	1.819 (2.078–3.070)	1.800 (1.114–2.908)
Q4	4.485 (3.115–6.458)	3.118 (1.936–5.024)	2.159 (1.295–3.602)	1.902 (1.219–2.968)
METS‐IR				
Q1	Ref.			
Q2	1.881 (1.199–2.951)	1.832 (0.998–3.361)	1.649 (0.895–3.038)	1.728 (0.989–3.020)
Q3	2.480 (1.638–3.756)	2.088 (1.148–3.796)	1.668 (0.913–3.045)	1.654 (0.930–2.943)
Q4	4.323 (2.843–6.573)	4.477 (2.453–8.170)	3.179 (1.682–6.008)	2.823 (1.643–4.851)

*Note:* Model1: unadjusted. Model2: model1 + Gender + Age + Race + Education + PIR. Model3: model2 + Alcohol + Uric Acid + Smoke + Folic Acid. Model4: model3 + Diabetes + Insulin + Triglyceride + HDL‐C + LDL‐C. Model 4 estimates should be interpreted with caution due to conceptual overlap between some adjusted variables and the IR indices.

### 3.3. Correlation Analysis of IR Substitutes and Gout Risk

**FIGURE 2:**
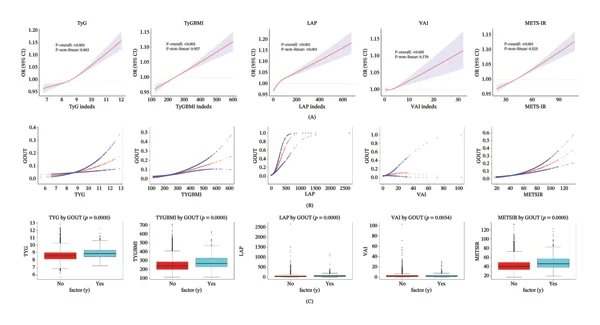
Correlation analysis of insulin resistance substitutes and gout risk. (A) Results of multivariate adjusted restricted cubic spline analysis. (B) Five insulin resistance surrogates and the smooth curve fitting for gout. (C) Boxplot; red represents nongout individuals and green represents gout patients.

Figure 2 presents the results of the correlation analysis of IR substitutes with the risk of gout, where Figure 2A presents the restricted cubic spline analysis results with OR values adjusted for all covariates, showing the dose–response relationship between the indices and gout prevalence. A nonlinear positive correlation was observed between gout risk and TyG and LAP (all *p* < 0.05), with no significant correlation with TyG‐BMI, VAI, and METS‐IR (all *p* > 0.05). LAP had the greatest effect, followed by METS‐IR. Figure 2B indicates the relationship between TyG, TyG‐BMI, LAP, VAI, and METS‐IR levels and gout risk. Each point represents the individual’s metabolic indicator level and gout risk. The figure contains two smoothed curves: a blue solid line for the overall trend and a red dashed line for a specific subgroup. The gout risk increased with increasing indicator levels, especially at TyG levels above 10. A box‐and‐whisker plot (Figure 2C) was drawn to analyze the correlation between IR‐related markers and gout. The levels of these markers were significantly higher in patients with gout than in nongout patients. These findings support the potential use of these biomarkers in assessing the risk of gout.

**FIGURE 3:**
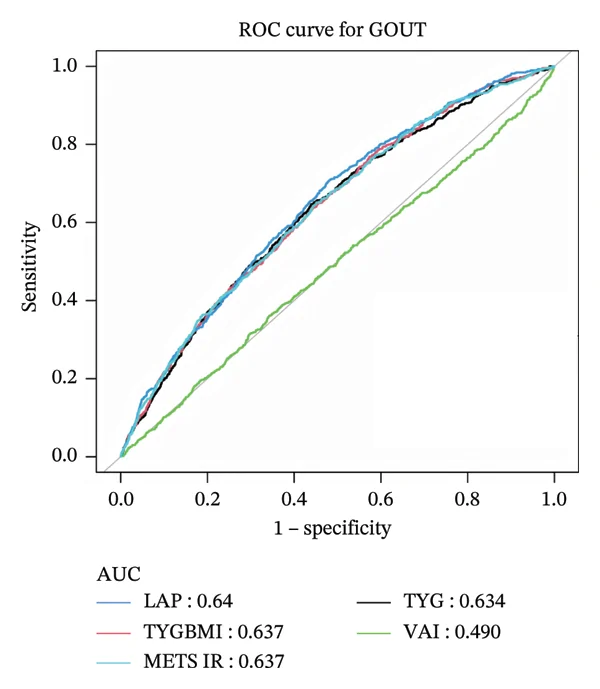
AUC for insulin resistance surrogates in relation to gout.

Figure 3 and Supporting Table 5 present the ROC analysis results and AUC of the five IR‐related markers for identifying gout after adjusting for covariates. Most markers had AUC values > 0.5 (except for VAI), indicating modest discriminative ability. LAP was the best marker among the indices examined, albeit with a modest AUC of 0.6478 (95% CI: 0.6263–0.6689). TyG‐BMI followed with an AUC of 0.6367 (95% CI: 0.6177–0.6568). METS‐IR combined with other factors had an AUC of 0.6358 (95% CI: 0.6158–0.6547). The AUC of the VAI was only 0.4844 (95% CI: 0.4617–0.5061), indicating poor discriminative ability. TyG had an AUC of 0.6292 (95% CI: 0.6106–0.6503), indicating modest performance. The optimal threshold, specificity, and sensitivity of these indices were evaluated. The optimal threshold for TyG‐BMI was 229.4128, specificity was 0.427, and sensitivity was 0.7686, while LAP had a threshold of 42.7828, specificity of 0.5202, and sensitivity of 0.7071. While these AUC values indicate that the indices are not suitable as standalone diagnostic tools, they may offer adjunctive information for identifying individuals with higher gout prevalence in primary care settings, though further prospective validation is needed before clinical application.

### 3.4. Results of Subgroup Analyses

Subgroup analyses stratified by clinically relevant factors were performed to evaluate potential effect modification (Supporting Tables 6–10). Significant interactions were observed for diabetes status with TyG and VAI (*p* for interaction < 0.05), where elevated TyG and VAI were associated with higher gout odds in nondiabetic individuals. Additionally, subgroup analyses stratified by SUA quartiles revealed that the associations of TyG‐BMI and METS‐IR with gout were most pronounced in individuals with the lowest SUA levels (*p* for interaction < 0.05). No significant interactions were observed for other stratification variables (all *p* for interaction > 0.05). Given the exploratory nature of these subgroup analyses and the potential for false‐positive findings due to multiple interaction testing, results should be interpreted cautiously (Supporting Table 11).

## 4. Discussion

This study revealed a significant correlation between the TyG index, TyG‐BMI, LAP, VAI, METS‐IR, and gout. After adjusting for covariates, all five IR‐related indices remained positively associated with gout across different models. When divided into quartiles, the risk of gout increased significantly with higher quartiles. High METS‐IR levels were significantly linked to gout risk (OR, 2.823; 95% CI: 1.643–4.851), followed by TyG‐BMI (OR, 2.290; 95% CI: 1.555–3.373), with similar patterns observed in most subgroups. These findings align with those of previous studies, suggesting that metabolic syndrome and IR play key roles in the pathogenesis [31, 32]. Some studies have reported a positive correlation between the TyG index and gout [20], and our results underscore its importance in assessing the risk of gout. This study extends these findings by introducing indices such as METS‐IR, TyG‐BMI, VAI, and LAP to offer a more comprehensive assessment of the association between metabolic dysfunction and gout.

METS‐IR, a novel IR indicator, was significantly associated with gout risk in this study, likely because of the combined effects of metabolic abnormalities, including obesity, hyperglycemia, and hyperlipidemia [33], which are closely related to uric acid metabolism. IR leads to decreased renal excretion of uric acid and increased SUA levels and gout [34]. Several biological pathways may underlie the association between IR and gout. First, IR has been suggested to impede uric acid renal excretion by enhancing sodium reabsorption in renal tubules [21, 35] and potentially affecting renal hemodynamics and tubular expression of uric acid transport proteins [21]. This could elevate SUA levels and increase the risk of gout. Second, IR is thought to trigger chronic inflammation via increased release of pro‐inflammatory cytokines, such as TNF‐α, IL‐1β, and IL‐6 [36]. These factors may exacerbate IR and promote urate crystal deposition in the joints, triggering gouty arthritis [37]. IR has also been linked to mitochondrial dysfunction and increased reactive oxygen species (ROS) production [36]. Increased ROS levels impair cellular function and affect uric acid metabolism and excretion, thereby potentially exacerbating hyperuricemia and gout risk [38]. A cross‐sectional study on UA in patients with type 2 diabetes mellitus and metabolic syndrome found that the overproduction of uric acid and reactive oxygen anion radicals may be key causes of IR [39]. While our study lacked direct measurements of these inflammatory cytokines to confirm such pathways, our subgroup analyses stratified by SUA quartiles provide an indirect epidemiological clue. Specifically, IR indices remained significantly associated with gout even in the lowest SUA stratum, suggesting that IR may contribute to gout risk through pathways that are at least partially independent of uric acid elevation (potentially including the aforementioned inflammatory or endothelial mechanisms). Nevertheless, given the cross‐sectional design of our study and the lack of direct validation using specific biomarkers, these mechanistic interpretations should be regarded as strictly hypothetical. They are intended to provide a conceptual framework for generating testable hypotheses, rather than definitive evidence of causal pathways. Future experimental and longitudinal studies with repeated urate measurements and serial biomarker assessments will be essential to confirm or refute these proposed mechanisms.

In our subgroup analyses, diabetes status significantly modified the associations of TyG and VAI with gout (P for interaction < 0.05), with stronger effects observed in nondiabetic individuals. This may be explained by the fact that diabetic patients often receive urate‐lowering medications that may mask metabolic risk, whereas nondiabetic individuals with elevated IR indices may exhibit compensatory hyperinsulinemia that drives renal urate reabsorption [40]. These findings suggest that TyG and VAI may be particularly informative for identifying gout risk in nondiabetic populations. However, given the multiple interaction tests performed, these results should be considered exploratory and warrant confirmation in future studies. Additionally, folate intake was inversely associated with gout risk, aligning with Mendelian randomization evidence [41]. This hypothesis‐generating finding suggests dietary factors may modulate IR pathways, warranting prospective validation.

This study identified that LAP exhibited the strongest discriminative ability among the examined indices, although its performance remained modest. These findings suggest that considering multiple metabolic indicators may provide additional information in gout risk assessment. Compared to SUA levels alone, the LAP index, which combines obesity and lipid indicators (WC and HDL‐C), reflects abdominal fat and dyslipidemia and serves as a crucial metabolic syndrome indicator [42]. This may better represent an individual’s metabolic status and its effect on gout. A large study in central China showed a dose–response link between LAP and the risk of hyperuricemia [43]. Epidemiological studies have associated hyperuricemia with obesity, indicating that centripetal obesity is a major contributor to hyperuricemia [44, 45]. These findings suggest that LAP may offer supporting information for gout risk stratification, though its clinical utility requires further investigation. The strong association between LAP and metabolic disorders, such as T2DM, is consistent with a potential role in gout pathophysiology. However, animal studies have shown that a high purine intake may indirectly promote gout via elevated METS‐IR [46]. Given the complex pathogenesis of gout, a composite index may offer more insight into patient health, especially for assessing metabolic health in centrally obese patients, aiding gout risk identification and assessment.

It should be noted that our different analytical approaches yielded complementary rather than contradictory findings. While METS‐IR indicated the strongest association in quartile‐based regression (OR = 2.823 for Q4 vs. Q1), LAP showed superior predictive discrimination in ROC analysis (AUC = 0.6478). These differences reflect distinct aspects of the indices′ performance: quartile regression emphasizes risk stratification at extreme values, whereas ROC analysis evaluates overall discriminatory capacity across all thresholds. Additionally, the nonlinear dose–response relationships observed for TyG and LAP suggest threshold effects in metabolic dysfunction, while METS‐IR showed a more linear pattern. One possible explanation is that METS‐IR’s composite nature (incorporating glucose, lipids, BMI, and HDL‐C) may produce more graded risk increases, whereas LAP’s focus on visceral adiposity may exhibit more pronounced threshold effects. Previous studies have also reported that LAP demonstrates nonlinear associations with gout and hyperuricemia [47], while METS‐IR shows consistent linear positive correlations with hyperuricemia‐related outcomes [48]. Additionally, comparative studies have shown that LAP and METS‐IR exhibit different predictive performance across metabolic outcomes [49]. However, this interpretation remains speculative and requires further investigation.

This is biologically plausible, as METS‐IR’s composite nature (incorporating glucose, lipids, BMI, and HDL‐C) may produce more graded risk increases, whereas LAP’s focus on visceral adiposity may exhibit more pronounced threshold effects.

This study has several strengths. It is a large sample to examine the association between these five IR alternatives and gout, potentially advancing related research. We used appropriate weights to ensure generalizability and adjusted for covariates to minimize confounding effects and increase the stability of the results. While our findings support the use of IR‐related indices in gout risk assessment, it’s important to consider other factors such as diet, genetic predisposition, and kidney function, which also play critical roles in gout etiology. Furthermore, this study had several limitations. First, the cross‐sectional design precludes causal inference, so the mechanisms, especially the stratified findings, should be viewed as hypothesis‐generating rather than conclusive. Second, gout was defined based on self‐reported physician diagnosis. Although SUA levels were higher in the gout group, this does not validate the diagnosis, as hyperuricemia is not synonymous with clinical gout. Importantly, the NHANES dataset lacks data on urate‐lowering therapy, crystal confirmation, clinical verification, and flare‐related characteristics, all of which would have refined case ascertainment. The absence of these variables likely introduces nondifferential misclassification, biasing our effect estimates toward the null. Third, detailed data on specific medication use (e.g., diuretics and insulin‐sensitizing agents), physical activity, and diet were lacking, potentially introducing residual confounding. However, adjusting for biomarkers such as SUA, glucose, and insulin in our models partially captured their indirect physiological effects. Finally, a major limitation is that the NHANES dataset did not include measurements of specific inflammatory cytokines; thus, we could not directly validate the inflammatory mechanisms linking IR to gout, and the related discussions remain speculative.

## 5. Conclusion

METS‐IR, TyG‐BMI, and LAP may serve as accessible, complementary indicators for identifying individuals with higher gout prevalence, though their modest discriminative performance precludes use as standalone diagnostic tools. Further validation in prospective studies is needed. This study offers advantages in terms of sample size and analytical methods and provides a comprehensive perspective. Future studies should explore the role of different metabolic markers in gout mechanisms to understand gout pathophysiology and develop targeted strategies.

**Nomenclature**
PIR: Family income‐to‐poverty ratio
HDL‐C: High‐density lipoprotein cholesterol
TG: Triglyceride
LDL‐C: Low‐density lipoprotein cholesterol
TyG: Triglyceride‐glucose index
TyG‐BMI: TyG index with body mass index
VAI: Visceral adiposity index
LAP: Lipid accumulation product
METS‐IR: Metabolic score for IR
95%CI: 95% confidence interval
ROC: Receiver operating characteristic
AUC: Area under the curves
VIF: Variance inflation factor
AIC: Akaike information criterion

## Author Contributions

Kongming Yang and Kewei Yang contributed to conduct research, data calculation, and article writing. Kongming Yang and Kewei Yang contributed to conceptualization, supervision, and editing. Kongming Yang, Yuhan Liu, Qin Wang, and Nan Zhang analyzed data. Kongming Yang and Yuhan Liu contributed to data collection. Yuanping Yin had primary responsibility for final content and designed the research and review. All authors reviewed the manuscript.

## Funding

No funding was received for this research.

## Conflicts of Interest

The authors declare no conflicts of interest.

## Supporting Information

Additional supporting information can be found online in the Supporting Information section.

## Supporting information


**Supporting Information 1** Supporting information 1: STROBE‐checklist‐v4‐cross‐sectional.

**Supporting Information 2** Supporting Table 1: Covariate adjustment strategy across models.

**Supporting Information 3** Supporting Table 2: Multicollinearity assessment of covariates.

**Supporting Information 4** Supporting Table 3: Model fit comparison by Akaike Information Criterion (AIC).

**Supporting Information 5** Supporting Table 4: Sensitivity analysis for insulin resistance surrogates in relation to gout.

**Supporting Information 6** Supporting Table 5: AUC for insulin resistance surrogates in relation to gout.

**Supporting Information 7** Supporting Table 6: Results of the subgroup analysis TyG.

**Supporting Information 8** Supporting Table 7: Results of the subgroup analysis TyG‐BMI.

**Supporting Information 9** Supporting Table 8: Results of the subgroup analysis LAP.

**Supporting Information 10** Supporting Table 9: Results of the subgroup analysis VAI.

**Supporting Information 11** Supporting Table 10: Results of the subgroup analysis METS‐IR.

**Supporting Information 12** Supporting Table 11: Baseline characteristics between the included (*n* = 14,582) and excluded (*n* = 45,260) populations.

## Data Availability

The data that support the findings of this study are publicly available in the National Health and Nutrition Examination Survey (NHANES) database at https://www.cdc.gov/nchs/nhanes/. We acknowledge the NHANES team for providing open‐access data.
