# Supplementary material for: Associations of Five Insulin Resistance‐Related Indices With Gout Risk in the 2007–2018 NHANES Cross‐Sectional Study
Source: Int J Endocrinol. 2026 Aug 29;2026:7431198. doi: 10.1155/ije/7431198 (PMC13525479; doi:10.1155/ije/7431198)
Supplement: Supplementary file 1 [file IJE-2026-7431198-s001.docx]

Supplementary Table 11

Baseline characteristics between the included (n = 14,582) and excluded (n = 45,260) populations

|  | **Level** | **Overall** | **Excluded** | **Included** | **P value** |
| --- | --- | --- | --- | --- | --- |
| Gender (%) | Female | 157941820.3 (51.1) | 107979387.4 (50.8) | 49962432.8 (51.8) | 0.052 |
|  | Male | 151091259.2 (48.9) | 104622723.0 (49.2) | 46468536.2 (48.2) |  |
| Age (mean (SD)) |  | 37.40 (22.42) | 32.68 (23.05) | 47.79 (16.80) | <0.001 |
| Race (%) | Mexican American | 32164172.9 (10.4) | 23790654.5 (11.2) | 8373518.3 ( 8.7) | <0.001 |
|  | Non-Hispanic Black | 37379274.6 (12.1) | 27282427.6 (12.8) | 10096847.0 (10.5) |  |
|  | Non-Hispanic White | 193393846.2 (62.6) | 128890758.5 (60.6) | 64503087.7 (66.9) |  |
|  | Other Hispanic | 19803318.7 ( 6.4) | 14001729.9 ( 6.6) | 5801588.8 ( 6.0) |  |
|  | Other Race - Including Multi-Racial | 26292467.2 ( 8.5) | 18636539.9 ( 8.8) | 7655927.3 ( 7.9) |  |
| Education (%) | Less than 9th grade | 12542757.8 ( 5.5) | 7082031.3 ( 5.4) | 5460726.5 ( 5.7) | 0.603 |
|  | 9-11th grade (Includes 12th grade with no diploma) | 23968601.3 (10.6) | 13615386.9 (10.5) | 10353214.4 (10.7) |  |
|  | High school graduate/GED or equivalent | 52204968.8 (23.0) | 30328076.4 (23.3) | 21876892.4 (22.7) |  |
|  | Some college or AA degree | 70747967.4 (31.2) | 40986186.8 (31.5) | 29761780.6 (30.9) |  |
|  | College graduate or above | 67053352.2 (29.6) | 38074997.0 (29.3) | 28978355.2 (30.1) |  |
| Smoke (%) | No | 129965371.5 (56.4) | 76520955.7 (57.1) | 53444415.8 (55.4) | 0.051 |
|  | Yes | 100466833.6 (43.6) | 57480280.4 (42.9) | 42986553.3 (44.6) |  |
| Alcohol drinks(day) (median [IQR]) |  | 2.00 [1.00, 3.00] | 2.00 [1.00, 3.00] | 2.00 [1.00, 3.00] | 0.1 |
