# Supplementary material for: Associations of Five Insulin Resistance‐Related Indices With Gout Risk in the 2007–2018 NHANES Cross‐Sectional Study
Source: Int J Endocrinol. 2026 Aug 29;2026:7431198. doi: 10.1155/ije/7431198 (PMC13525479; doi:10.1155/ije/7431198)
Supplement: Supplementary file 2 [file IJE-2026-7431198-s004.docx]

Supplementary Table 5. AUCs for Insulin Resistance Surrogates in Relation to Gout

| Variables | AUC (95% CI) | Best threshold | Specificity | Sensitivity |
| --- | --- | --- | --- | --- |
| TYG+ other factors | 0.6292(0.6106-0.6503) | 8.6481 | 0.5607 | 0.6471 |
| TYGBMI+ other factors | 0.6367(0.6177-0.6568) | 229.4128 | 0.427 | 0.7686 |
| LAP+ other factors | 0.6478(0.6263-0.0.6689) | 42.7828 | 0.5202 | 0.7071 |
| VAI+ other factors | 0.4844(0.4617-0.5061) | 1.6564 | 0.659 | 0.36 |
| METS-IR+ other factors | 0.6358(0.6158-0.6547) | 42.5492 | 0.5488 | 0.6514 |

Notes:Other factors: Gender, Age, Race, Education, PIR, Alcohol, Uric Acid, Smoke, Folic , Diabetes, Insulin, Triglyceride, HDL-C, LDL-C.

Abbreviations: AUC, area under the curve, TyG, triglyceride-glucose index; TyG-BMI, TyG index with body mass index; LAP, lipid accumulation product; VAI, visceral adiposity index; METS-IR, metabolic score for IR.
