# Supplementary material for: Associations of Five Insulin Resistance‐Related Indices With Gout Risk in the 2007–2018 NHANES Cross‐Sectional Study
Source: Int J Endocrinol. 2026 Aug 29;2026:7431198. doi: 10.1155/ije/7431198 (PMC13525479; doi:10.1155/ije/7431198)
Supplement: Supplementary file 3 [file IJE-2026-7431198-s006.docx]

Supplementary Table 3. Model Fit Comparison by Akaike Information Criterion (AIC)

| **IR Index** | **Model 1 AIC** | **Model 2 AIC** | **Model 3 AIC** | **Model 4 AIC** | **Best Model** | **Min AIC** |
| --- | --- | --- | --- | --- | --- | --- |
| TyG | 4879.10 | 4475.90 | 4330.11 | 4307.70 | Model 4 | 4307.70 |
| TyG-BMI | 4831.29 | 4386.91 | 4274.88 | 4265.97 | Model 4 | 4265.97 |
| VAI | 4979.52 | 4491.34 | 4332.59 | 4305.12 | Model 4 | 4305.12 |
| LAP | 4838.92 | 4432.69 | 4311.53 | 4290.08 | Model 4 | 4290.08 |
| METS-IR | 4845.82 | 4397.58 | 4284.49 | 4274.39 | Model 4 | 4274.39 |
