# Supplementary material for: Associations of Five Insulin Resistance‐Related Indices With Gout Risk in the 2007–2018 NHANES Cross‐Sectional Study
Source: Int J Endocrinol. 2026 Aug 29;2026:7431198. doi: 10.1155/ije/7431198 (PMC13525479; doi:10.1155/ije/7431198)
Supplement: Supplementary file 4 [file IJE-2026-7431198-s007.docx]

Supplementary Table 2. Multicollinearity Assessment of Covariates

| Covariates | Variance Inflation Factor (VIF) |
| --- | --- |
| Gender | 1.17 |
| Age | 1.14 |
| Race | 1.04 |
| Education | 1.06 |
| PIR | 1.14 |
| Alcohol | 1.00 |
| Smoke | 1.06 |
| Folic Acid | 1.02 |
| Uric Acid | 1.16 |
| Diabetes | 1.12 |
| Insulin | 1.06 |
| Triglyceride | 1.29 |
| HDL-C | 1.22 |
| LDL-C | 1.05 |
