# Supplementary material for: Associations of Five Insulin Resistance‐Related Indices With Gout Risk in the 2007–2018 NHANES Cross‐Sectional Study
Source: Int J Endocrinol. 2026 Aug 29;2026:7431198. doi: 10.1155/ije/7431198 (PMC13525479; doi:10.1155/ije/7431198)
Supplement: Supplementary file 5 [file IJE-2026-7431198-s008.docx]

Supplementary Table 1. Covariate adjustment strategy across models

| Model | Adjustment variables |
| --- | --- |
| Model 1 | Unadjusted |
| Model 2 | Gender, Age, Race, Education, PIR |
| Model 3 | Model 2 +Alcohol+Uric Acid+Smoke+Folic Acid |
| Model 4 (primary) | Model 3 +Diabetes+Insulin+Triglyceride+HDL-C+LDL-C |
| Sensitivity analysis | Gender, Age, Race, Education, PIR, Alcohol, Smoke, Folic Acid, Diabetes, LDL-C,eGFR, and serum creatinine |
