# Supplementary material for: Associations of Five Insulin Resistance‐Related Indices With Gout Risk in the 2007–2018 NHANES Cross‐Sectional Study
Source: Int J Endocrinol. 2026 Aug 29;2026:7431198. doi: 10.1155/ije/7431198 (PMC13525479; doi:10.1155/ije/7431198)
Supplement: Supplementary file 7 [file IJE-2026-7431198-s012.docx]

Supplementary Table 4. Sensitivity analysis for Insulin Resistance Surrogates in Relation to Gout

| **IR Index** | **Q1 (Ref.)** | **Q2 (OR, 95% CI)** | **Q3 (OR, 95% CI)** | **Q4 (OR, 95% CI)** |
| --- | --- | --- | --- | --- |
| TyG | Ref. | 1.052 (0.690, 1.603) | 1.427 (0.904, 2.251) | 2.120 (1.364, 3.295) |
| TyG-BMI | Ref. | 1.462 (1.021, 2.096) | 2.067 (1.496, 2.855) | 3.912 (2.788, 5.488) |
| VAI | Ref. | 1.204 (0.847, 1.711) | 1.514 (1.053, 2.178) | 2.229 (1.550, 3.206) |
| LAP | Ref. | 1.466 (0.961, 2.234) | 2.305 (1.627, 3.267) | 3.330 (2.254, 4.921) |
| METS-IR | Ref. | 1.422 (0.900, 2.247) | 1.838 (1.256, 2.689) | 3.577 (2.328, 5.496) |

Notes: Adjusted for Gender, Age, Race, Education, PIR, Alcohol, Smoke, Folic Acid, Diabetes, LDL-C,eGFR, and serum creatinine.
